# Lysine Residues in the MK-Rich Region Are Not Required for Binding of the PbsP Protein From Group B Streptococci to Plasminogen

**DOI:** 10.3389/fcimb.2021.679792

**Published:** 2021-09-08

**Authors:** Francesco Coppolino, Letizia Romeo, Giampiero Pietrocola, Germana Lentini, Giuseppe Valerio De Gaetano, Giuseppe Teti, Roberta Galbo, Concetta Beninati

**Affiliations:** ^1^Department of Biomedical, Dental and Imaging Sciences, University of Messina, Messina, Italy; ^2^Department of Human Pathology and Medicine, University of Messina, Messina, Italy; ^3^Department Molecular Medicine, Biochemistry Section, University of Pavia, Pavia, Italy; ^4^Charybdis Vaccines Srl, Messina, Italy; ^5^Department of Chemical, Biological, Pharmaceutical and Environmental Sciences, University of Messina, Messina, Italy; ^6^Scylla Biotech Srl, Messina, Italy

**Keywords:** *Streptococcus agalactiae*, MK-rich domain, plasminogen, cell wall-proteins, adhesion molecules

## Abstract

Binding to plasminogen (Plg) enables bacteria to associate with and invade host tissues. The cell wall protein PbsP significantly contributes to the ability of group B streptococci, a frequent cause of invasive infection, to bind Plg. Here we sought to identify the molecular regions involved in the interactions between Plg and PbsP. The K4 Kringle domain of the Plg molecule was required for binding of Plg to whole PbsP and to a PbsP fragment encompassing a region rich in methionine and lysine (MK-rich domain). These interactions were inhibited by free L-lysine, indicating the involvement of lysine binding sites in the Plg molecule. However, mutation to alanine of all lysine residues in the MK-rich domain did not decrease its ability to bind Plg. Collectively, our data identify a novel bacterial sequence that can interact with lysine binding sites in the Plg molecule. Notably, such binding did not require the presence of lysine or other positively charged amino acids in the bacterial receptor. These data may be useful for developing alternative therapeutic strategies aimed at blocking interactions between group B streptococci and Plg.

## Introduction

A wide variety of bacterial species are capable of interacting with plasminogen (Plg), a process that is thought to enhance their ability to colonize and invade host tissues (Lottenberg et al., 1994; Boyle and Lottenberg, 1997; Lahteenmaki et al., 2001; Walker et al., 2005; Sanderson-Smith et al., 2012; Fulde et al., 2013). Plg is produced in the liver and is released in plasma, where it reaches elevated concentrations (around 200 mg/l or 2 μM). Lower amounts of this protein are associated with various types of tissues, in which Plg is found predominantly on the cell surface and in the extracellular matrix. The presence of Plg on the surface of host cells can enhance microbial adherence, as shown using epithelial cells (Papasergi et al., 2010; Bhattacharya et al., 2012; Agarwal et al., 2013). Moreover, soluble Plg can be recruited from plasma or exudates to microbial surfaces to be converted to plasmin (Pl), an active form of the molecule endowed with potent protease activity (Castellino and Ploplis, 2005). By this mechanism, surface-associated plasmin can contribute to degradation of the extracellular matrix and fibrin barriers, leading to tissue disruption and hematogenous dissemination to distant organs, such as the brain (Bhattacharya et al., 2012; Peetermans et al., 2016).

The mature circulating form of Plg (791 amino acids, 93 kDa) bears an Activation Peptide (AP) domain followed by 5 Kringle domains (K1–K5, each displaying three loops and three intra-domain disulfide bridges) and by the S1 Peptidase Domain (SPD) at the C-terminal region (Claeys et al., 1976; Castellino and Powell, 1981). The Kringle domains of Plg are able to interact *via* their lysine-binding sites (LBS) with multiple ligands, including host Plg receptors/targets, such as fibrin and α2 anti-plasmin, and bacterial proteins (Castellino and McCance, 1997; Bhattacharya et al., 2012). The Kringle LBS interact not only with lysine residues present in Plg-binding proteins, but also with free L-lysine and with the analogous zwitterionic ligand ϵ-aminocaproic acid (EACA). EACA inhibits fibrinolysis by competing with fibrin for binding to LBS in the Pl molecule and is used to treat acute bleeding disorders. Structural analysis of Kringle domains indicates that LBS consist of shallow surfaces bearing a dipole in which the opposite charges are separated by a hydrophobic region of highly conserved aromatic residues (Tulinsky et al., 1988). LBS in Kringle domains bind free lysine and EACA in the following order of affinity: K1 > K4 > K5 > K2 (Lin et al., 2000; Sun et al., 2002) while the K3 domain displays only slight lysine-binding activity. Streptokinase and staphylokinase are extracellularly secreted products of, respectively, *Streptoccoccus pyogenes* and *Staphylococcus aureus* that directly bind and activate Plg (Verhamme and Bock, 2014; Verhamme et al., 2015; Nguyen and Vogel, 2016; Rafipour et al., 2019). Other extracellular proteins, such as Skizzle from GBS, and surface-associated Plg-binding bacterial products (often referred to as bacterial Plg receptors), rely instead on external activators, such as host-derived uPA or tPA, for conversion of bound Plg into Pl (Wiles et al., 2010; Peetermans et al., 2016). Bacterial Plg receptors include “moonlighting” cytoplasmic enzymes, such as α-enolase and glyceraldehyde-3-phosphate dehydrogenase (GAPDH) (Winram and Lottenberg, 1996; Pancholi and Fischetti, 1998; Bergmann et al., 2003; Seifert et al., 2003; Boël et al., 2005) as well as specialized lipoproteins or cell wall proteins, such as Plg-binding streptococcal M- and M-like proteins (Berge and Sjobring, 1993; Wistedt et al., 1995; Ringdahl and Sjobring, 2000; Bhattacharya et al., 2012). A common feature of bacterial surface Plg receptors is their ability to interact with LBS, as indicated by inhibition of these interactions using free lysine or EACA.

*Streptococcus agalactiae* (also named group B *Streptococcus* or GBS) is a gram-positive encapsulated bacterium that can behave as a commensal of the human gastrointestinal and genital tracts or as an agent of invasive infections. Clinical manifestations of GBS disease include sepsis and meningitis in neonates and an increasing variety of conditions in adults with predisposing factors, in pregnant women and in the elderly (Skoff et al., 2009; Edmond et al., 2012; Le Doare and Heath, 2013). Plg binding and acquisition of plasmin-mediated proteolytic activity by GBS have a crucial role in its ability to spread hematogenously to the brain and other organs (Magalhaes et al., 2013). Plg binding by GBS is at least partially mediated by PbsP (standing for Plasminogen binding surface Protein), a cell-wall-anchored protein of 521-aa. PbsP contains an N-terminal domain, two 150-aa SSURE repeat domains (Bumbaca et al., 2004), a methionine and lysine-rich (MK-rich) region, and a cell wall-anchoring LPxTG motif (Buscetta et al., 2016). PbsP is involved in hematogenous dissemination and blood brain barrier invasion by GBS (Buscetta et al., 2016; Lentini et al., 2018) and is an interesting vaccine candidate, because of its high degree of conservation among GBS clinical isolates and its strong upregulation *in vivo* (Cook et al., 2018; Lentini et al., 2018). Moreover, PbsP is a multifunctional adhesin capable of also binding vitronectin, which might contribute to its ability to promote GBS adherence to epithelial cells (De Gaetano et al., 2018). In view of its importance in pathogenesis, we sought in the present study to obtain insights into the molecular regions responsible for PbsP binding to Plg. It was found that such interactions require a specific sequence in the MK-rich domain of PbsP and the Kringle 4 LBS of Plg. However, the presence of lysine or other positively charged amino acid residues in the MK-rich region was not required for binding of this region to Plg. These data may be useful to develop therapeutic strategies aimed at preventing GBS interactions with Plg and to better understand binding of bacterial receptors to Plg.

## Materials and Methods

### Peptides, Recombinant Fragments and Antibodies

The recombinant PbsP (rPbsP), SSURE domains (rSSURE-1+2 and rSSURE-2) and rMK-rich domain used in this study were produced as described (Garibaldi et al., 2010; Papasergi et al., 2010; Buscetta et al., 2014; Buscetta et al., 2016). The peptides indicated as Fr1, Fr2, Fr3 and Fr4, encompassing the MK-rich domain, were purchased from GenScript Itd (Hong Kong).

To obtain the M12K fragment and its mutated forms designated Mut1, Mut2, Mut3 and Mut4 (**Supplementary Table 1**), the corresponding oligonucleotides were obtained by ATG:biosynthetics GmbH (Germany). After amplification with the primers described in **Supplementary Table 2**, PCR products were cloned into a Gateway pDONR221 vector (Thermo Fisher Scientific, Waltham, Massachusetts) according to the manufacturer’s instructions. Next, the recombinant purified plasmids were used to obtain expression vectors pDEST15_M12K, pDEST15_Mut1, pDEST15_Mut2, pDEST15_Mut3, and pDEST15_Mut4. Transformed *E. coli* BL21 (DE3) strains were grown to purify the peptides as fusions to Gluthatione S-Transferase, as previously described (Buscetta et al., 2016). Polyclonal antiserum against purified Plg was raised in rabbits using purified human Plg as an antigen. Purification of rabbit antibodies from sera was performed by affinity chromatography using protein G-Sepharose columns (GE Healthcare, Buckinghamshire, UK). Goat anti-rabbit IgG horseradish peroxidase (HRP)-conjugated secondary antibody was purchased from Dako Cytomation (Glostrup, Denmark). Unless stated otherwise, all other reagents were purchased from Sigma-Aldrich (St. Louis, Missouri).

### Plasminogen and Kringle Domains

Human plasma was obtained from healthy volunteers with informed consent and permission of the ethical board of the University of Pavia (permit no. 19092013). After centrifugation, the plasma fraction was frozen in aliquots and stored at −20°C (Pietrocola et al., 2016). Plasminogen was purified from plasma by affinity chromatography on a Lys-Sepharose column (Deutsch and Mertz, 1970). Kringle 1–3 (P1667) and Kringle 1–4 (MBS634949) were purchased from Sigma-Aldrich and MyBiosource (San Diego, California), respectively. Mini-Plg (residues Val_442_-Asn_790_) was obtained by digestion of Plg with porcine pancreatic elastase (Sigma-Aldrich), as previously described (Váli and Patthy, 1982; Christensen and Mølgaard, 1992).

### ELISA, Dot and Western Blot Assays

Binding of Plg or its fragments to recombinant proteins (rPbsP, rSSURE-1+2, rSSURE-2, rMK-rich, M12K, Mut1, Mut2, Mut3 and Mut4) was determined by ELISA, dot and Western blot assays as described (Pietrocola et al., 2016). For ELISA assays, microtiter wells were coated a 4°C with equimolar amounts of rPbsP or PbsP fragments. Blocking of the wells was done for 1h at room temperature (RT) with 200 µl of 4% bovine serum albumin (BSA, Sigma-Aldrich) in PBS, followed by the addition of Plg, K 1-3, K 1-4 or Mini-Plg. The plates were then incubated with rabbit polyclonal anti-Plg (1:2000 in PBS 0,1% BSA) and anti-rabbit horseradish peroxidase-conjugated IgG (diluted 1:10000 in PBS 0,1% BSA). Plates were developed with o-phenylenediamine dihydrochloride (OPD) and the absorbance (490 nm) was determined using an ELISA plate reader. For competitive ELISA, plates were coated and blocked as above and incubated for 1h at RT with rPbsP or PbsP fragments in the presence of the indicated inhibitors. In selected experiments, plates were sensitized with rPbsP or its fragments, probed with Plg, K 1-3, K 1-4 or Mini-Plg and developed, with rabbit polyclonal anti-Plg, followed by anti-rabbit horseradish peroxidase-conjugated IgG.

## Results

### The Kringle 4 Domain of Plg Is Required for Binding to PbsP

**Figure 1:**
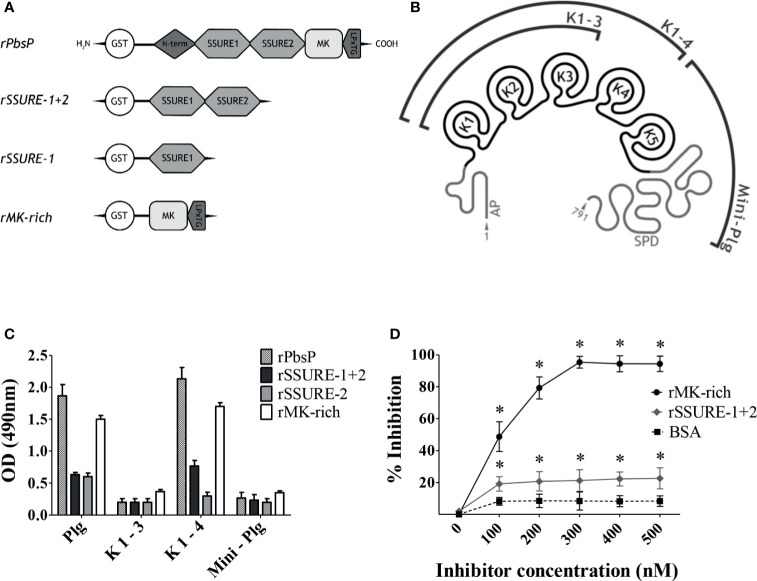
The Kringle 4 domain of Plg is required for binding to PbsP. **(A)**, Schematic representation of recombinant Plasminogen binding surface Protein (PbsP) and its derived recombinant fragments. **(B)**, Structure of human Glu-Plasminogen and its fragments: AP domain, K 1-3 encompassing Kringle 1 to 3, K 1-4 encompassing Kringle 1 to 4 and Mini-Plg encompassing Kringle 5 and S1 Peptidase Domain (SPD). **(C)**, ELISA for selective binding of PbsP and its fragments to Plg and its derivatives. An equimolar amount of rPbsP and its derived fragments (250 nM) was immobilized on the surface of microtiter wells, and their binding was tested with Plg, K1-3, K1-4, or Mini-Plg (100 nM). Complexes were detected with polyclonal anti-Plg antibodies followed by HRP-conjugated goat anti-rabbit IgG. **(D)**, Selective inhibition of PbsP-Plg interactions. Competitive ELISA assays were done with immobilized rPbsP (250 nM) to which Plg (100 nM) was added in the presence of increasing concentrations of rSSURE-1+2 or rMK-rich fragments. BSA (bovine serum albumin) was used as a control inhibitor. Inhibition ability is shown as percentage. Data are means ± SD from three independent experiments conducted in duplicate. *p <0.05 as determined by Wilcoxon rank sum test analysis.

In order to identify the molecular regions involved in interactions between PbsP and Plg, we analyzed the binding of isolated recombinant PbsP domains to different Plg fragments by ELISA. In these assays we used the whole PbsP protein, the MK-rich domain and 2 different SSURE domain fragments (rSSURE-1+2 and rSSURE-2; **Figure 1A**). These products were immobilized on plates and probed with the whole Plg protein or three different Plg fragments encompassing Kringle 1 to 3, Kringle 1 to 4 and Mini-Plg (**Figure 1B**). Under these conditions, the MK-rich domain bound Plg almost as efficiently as the whole PbsP protein (**Figure 1C**). In contrast, the SSURE domains showed only moderate, albeit significant, binding to Plg. Notably, both the MK-rich domain and whole PbsP bound the Plg fragment encompassing Kringle 1 to 4, but not other fragments, indicating that the Kringle 4 domain is required for PbsP-Plg interactions. To further study the role of the MK-rich domain, we tested the ability of the isolated MK-rich fragments to compete with whole PbsP for binding to Plg. The whole protein was adsorbed onto microtiter plates and Plg binding was assessed in the presence of inhibitors. As shown in **Figure 1D**, the addition of the MK-rich domain produced a dose-dependent inhibition in the binding of Plg to PbsP and could almost completely abrogate such binding at the highest doses, while rSSURE-1+2 was only partially effective as an inhibitor. Collectively these data indicate that the Plg-binding ability of PbsP predominantly resides in its MK-rich domain and requires the Kringle 4 domain.

### Free L-Lysine Inhibits Interactions Between Plg and the MK-Rich Domain

**Figure 2:**
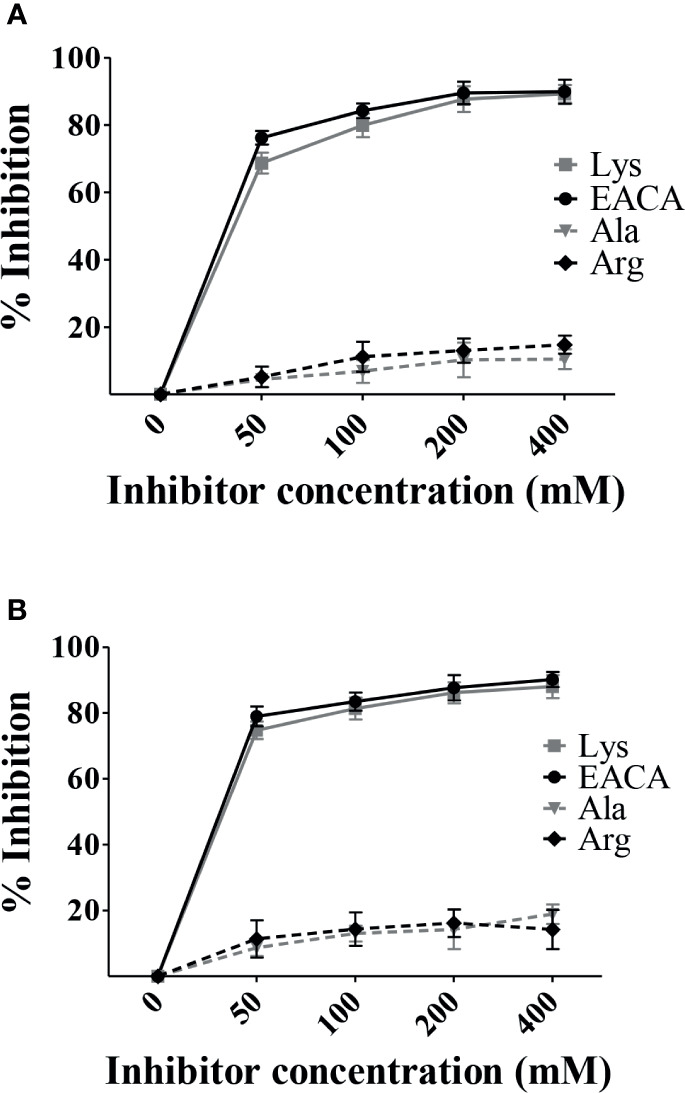
Free L-lysine inhibits interactions between Plg and the MK-rich domain. **(A, B)**, Selective inhibition of rMK-rich/rPbsP-Plg interactions. Competitive ELISA assays were done with immobilized rMK-rich or rPbsP (250 nM) to which Plg (100 nM) was added in the presence of increasing concentrations of ε-aminocaproic acid (EACA) or L-lysine (Lys). L-alanine (Ala) and L-arginine (Arg) were used as negative controls. Inhibition ability is shown as percentage. Data are means ± SD from three independent experiments conducted in duplicate.

In order to identify the molecular regions involved in PbsP-Plg interactions, we next investigated the involvement of LBS sites in binding of the MK-rich fragment to Plg by testing the inhibitory effects of soluble L-lysine. To this end, Plg binding to immobilized PbsP or its fragments was tested in the presence of soluble L-lysine or its analog EACA, which is frequently used to probe the interactions between LBS and Plg-binding proteins (Lin et al., 2000). Under these conditions, both free lysine and EACA produced a dose-dependent inhibition of Plg binding to the MK-rich fragment (**Figure 2A**). In contrast, alanine (a non charged amino acid) and arginine (a charged amino acid), used as controls, were totally ineffective. As expected, similar results were obtained when assessing the ability of lysine and EACA to inhibit Plg binding of whole PbsP (**Figure 2B**). Collectively these data suggest that LBS are involved in interactions between Plg and the MK-rich domain of PbsP.

### A C-Terminal Fragment of the MK-Rich Domain Is Sufficient for Binding to Plg

**Figure 3:**
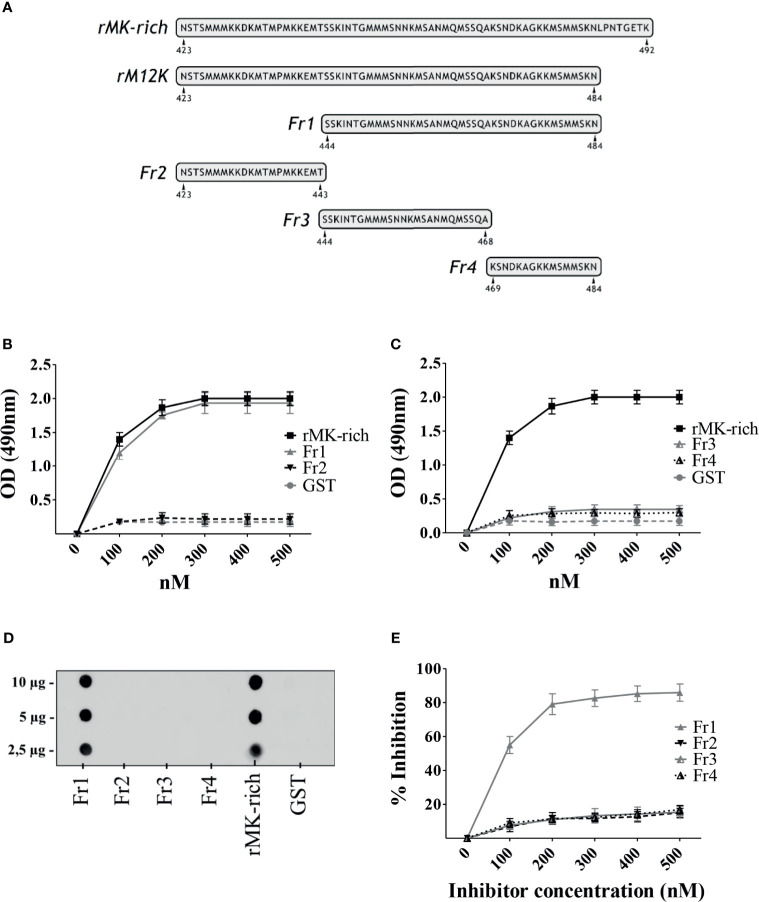
A C-terminal fragment of the MK-rich domain is sufficient for binding to Plg. **(A)**, Schematic representation and sequence of truncated forms of MK-rich domain. **(B, C)**, Binding of MK-rich and its truncated forms to Plg. Recombinant MK-rich and its derived fragments (250 nM) were immobilized on the surface of microtiter wells, and their binding was tested with increasing concentrations of Plg. Bound Plg was detected with rabbit antibodies to human Plg followed by HRP-conjugated goat anti-rabbit IgG. **(D)**, Dot blot analysis of MK-rich/fragments-Plg interactions. Increasing concentrations of rMK-rich or its fragments were spotted onto the nitrocellulose membranes and probed using 1μg of Plg, which were detected using anti‐Plg antibodies. GST was used as negative control. Shown are data from one representative experiment of two producing similar results. **(E)**, Selective inhibition of MK-rich-Plg interactions. Competitive ELISA assays were done with immobilized rMK-rich (250 nM) to which Plg (100 nM) was added in the presence of increasing concentrations of Fr1, Fr2, Fr3 and Fr4. Inhibition ability is shown as percentage. Data are means ± SD from three independent experiments conducted in duplicate.

In view of the significant Plg-binding activities of the MK-rich domain of PbsP, further studies focused on the mechanisms of such interactions. In order to identify the minimal region of the MK-rich domain that still maintains reactivity with Plg, we produced truncated forms of this domain. First we produced an N-terminally truncated form (designated Fr1) spanning amino acids S_444_ to N_484,_ and a shorter fragment (designated Fr2) spanning the remaining part of the domain (N_423_ to T_443_; **Figure 3A**). When tested by ELISA and dot blot analysis, Fr1 fully retained the ability of the entire MK-rich domain to bind Plg, while Fr2 was inactive (**Figures 3B, D**). Next, we produced a truncated form of Fr1, designated Fr4, (K_469_-N_484_) lacking the 25 N-terminal amino acids and Fr3, the complementary fragment, spanning the 25 N-terminal amino acids (S_444_-A_468_). As shown in **Figures 3C, D**, neither of these fragments bound Plg to any extent. In addition, assays using Fr2, Fr3 and Fr4 as inhibitors indicated that none of these fragments inhibited binding of Plg to the MK-rich domain (**Figure 3E**). In contrast, as expected, Fr1 showed strong inhibitory activities under the same conditions (**Figure 3E**). These results indicate that the 21 N-terminal amino acids (N_423_ to T_443_) of the MK-rich region are not required for Plg binding and that Fr1, but not shorter fragments, recapitulates the ability of the MK-rich domain to bind Plg.

### Lysine Residues in the MK-Rich Domain Are Not Involved in Plg Binding

**Figure 4:**
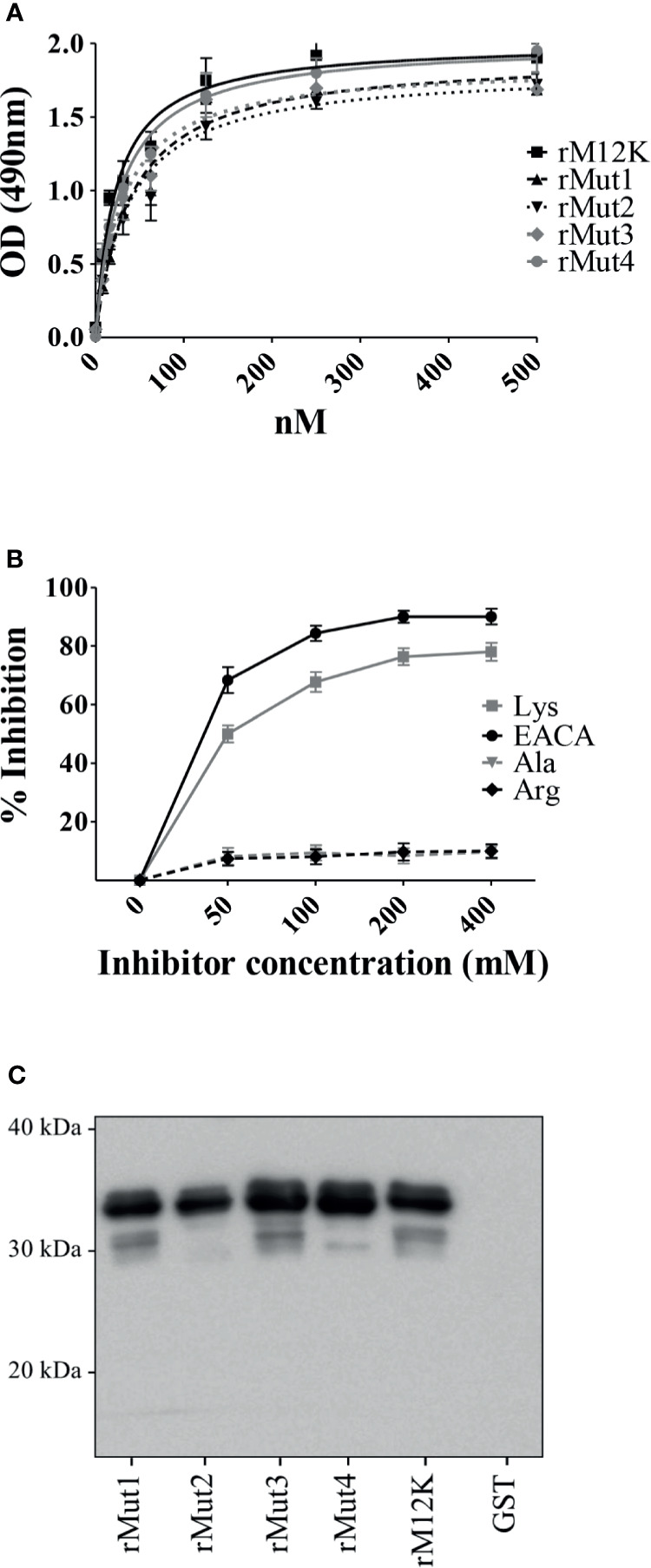
Lysine residues in the MK-rich domain are not involved in Plg binding. **(A)**, Binding of M12K and its mutated forms to Plg. Recombinant M12K and its mutated forms (250 nM) were immobilized on the surface of microtiter wells, and their binding was tested with increasing concentrations of Plg. Bound Plg was detected with rabbit anti-Plg antibodies and HRP-conjugated goat anti-rabbit IgG. **(B)**, Selective inhibition of Mut4-Plg interactions. Competitive ELISA assays were done with immobilized rMut4 (250 nM) to which Plg (100 nM) was added in the presence of the indicated concentrations of (EACA) or L-lysine (Lys). L-alanine (Ala) and L-arginine (Arg) were used as negative controls. Inhibition ability is shown as percentage. Data are means ± SD from three independent experiments conducted in duplicate. **(C)**, Western blot analysis of binding to Plg of M12K and its mutated forms. Recombinant M12K or its mutated forms were loaded onto SDS-PAGE (5µg/lane), transferred to nitrocellulose membrane and probed with Plg (1µg/ml). Bound Plg was detected using a primary polyclonal anti-Plg antibody followed by secondary HRP-conjugated goat anti-rabbit IgG. GST was used as negative control. Numbers indicate the molecular mass of protein standards in kDa. Shown are data from one representative experiment of two producing similar results.

Previous studies have demonstrated that terminal or internal lysine residues in a number of bacterial Plg receptors are required for optimal interactions with Plg (Bergmann et al., 2001; Derbise et al., 2004; Wiles et al., 2010; Moreau et al., 2017; Nagarajan et al., 2019). In view of the ability of L-lysine to inhibit interactions between the MK-rich domain and Plg, as shown above, we hypothesized that LBS in the Plg molecule interact with one or more of the lysine residues that are abundantly present in the MK-rich domain. To verify this hypothesis, we produced several mutated forms of the MK-rich domain in which different lysine residues were replaced by alanine (**Supplementary Table 1**). Next, we used these mutated forms to assess their binding to Plg by ELISA and Western blot analysis. Surprisingly, none of the mutated forms showed decreased binding to Plg as compared with the corresponding fragment bearing all lysine residues and designated as M12K (**Figures 4A, C**). In particular, an MK-rich fragment in which all lysine residues were replaced by alanine (rMut4) bound Plg as efficiently as the wild-type form of the molecule (**Figures 4A, C**). Next, it was of interest to ascertain whether the rMut4 fragment in which all lysine residues were replaced by alanine could still be inhibited by free lysine in its interactions with Plg. **Figure 4B** shows that this was indeed the case, since both L-lysine and EACA inhibited Plg binding by the rMut4 fragment. These data indicate that lysine residues in the MK-rich region are not required for interactions between this region and the LBS in the Plg molecule.

**Figure 5:**
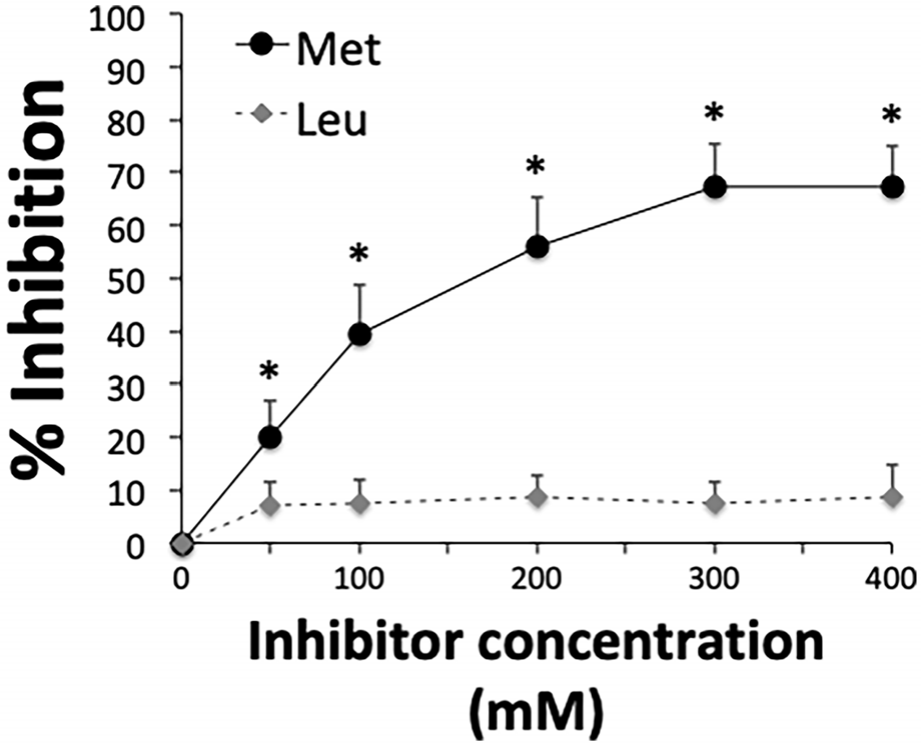
Free L-methionine inhibits interactions between Plg and the MK-rich domain. Selective inhibition of the interactions between Plg and the rMut4 fragment that lacks all of the lysine residues present in the MK-rich fragment. Competitive ELISA assays were done with immobilized rMut4 fragment (250 nM) to which Plg (100 nM) was added in the presence of increasing concentrations of L-methionine (Met). L-leucine (Leu) was used as a negative control. Inhibition ability is shown as percentage. Data are means ± SD from three independent experiments conducted in duplicate. *p < 0.05 as determined by Wilcoxon rank sum test analysis.

A remarkable feature of the MK-rich region is the abundant presence of methionine, which accounts for 26% of all amino acid residues (**Figure 3A**). Since methionine is a nonpolar amino acid, we hypothesized that hydrophobic interactions are involved in binding of the MK-rich region to Plg LBS, which contain pockets of hydrophobic residues. To explore the possible involvement of methionine residues in binding of the MK-rich region to Plg, we tested the effects of increasing concentrations of free L-methionine on this interaction. The presence of methionine at 50 mM or higher concentrations significantly, albeit partially, inhibited binding of the rMut4 fragment to Plg (**Figure 5**), suggesting the possible involvement of methionine residues in such binding.

## Discussion

Our previous studies have indicated that the cell wall protein PbsP significantly contributes to the ability of GBS to bind plasminogen and acquire plasmin-dependent protease activity (Buscetta et al., 2016). *In vivo*, PbsP plays a major role in brain invasion by GBS, a process that is at least partially mediated by surface plasmin activity (Buscetta et al., 2016; Lentini et al., 2018). Therefore, better understanding of the molecular mechanisms by which PbsP binds plasminogen may lead to the development of adjunctive methods to control GBS disease. In the first part of our present study we sought to identify the molecular region of Plg involved in binding to PbsP. It was found that such binding was inhibited by free L-lysine suggesting the involvement of LBS in one or more Kringle domains of Plg. In this respect, our data resemble observations conducted with a wide variety of other bacterial Plg receptors, whose interactions with Plg are lysine- or EACA-inhibitable (Lahteenmaki et al., 2001; Bergmann et al., 2011; Bhattacharya et al., 2012; Sanderson-Smith et al., 2012; Peetermans et al., 2016). However, relatively few studies have investigated the specific type of LBS or Kringle domain involved. Plg-binding M proteins from *Streptococcus pyogenes* selectively interact with K2, which contains a low-affinity LBS (Wistedt et al., 1998; Fu et al., 2008), while SCM, an M-like protein from *Streptococcus canis*, binds the K5 of Plg and Mini-Plg (Fulde et al., 2011). The LenA protein from *Leptospira interrogans* was found to bind to a Plg fragment spanning K1 to K3 (Verma et al., 2010). To our knowledge, specific binding of bacterial receptors to the K4 domain of Plg has not been previously reported.

We found here that PbsP and its MK-rich region bound to a polypeptide encompassing the K1-4 Plg domains but not to a shorter fragment encompassing K1-3, indicating that the K4 domain is required for binding. It is likely that this requirement reflects direct binding of the MK-rich region to the K4 domain rather than indirect effects of the K4 domain in changing the conformation of K1-3 in a way that promotes binding of these domains to Plg. In fact, Kringles are considered functionally and structurally independent domains that are highly stabilized by internal bonds, including disulfide bridges (Mulichak et al., 1991; De Vos et al., 1992). However, further studies involving isolated Kringle domains will be needed to formally demonstrate binding of the MK-rich region to K4. Collectively our data and those of previous investigations suggest a high degree of specificity in the binding to distinct LBS of the various bacterial proteins studied thus far, likely reflecting considerable differences in the Plg-binding motifs of the various receptors.

In the second part of our study we sought to identify the molecular region(s) of PbsP involved in binding to Plg. In agreement with previous studies (Buscetta et al., 2016), we found that both the SSURE domains and the MK-rich domain (i.e. a PbsP region rich in methionine and lysine) were capable of binding Plg and that the latter domain was highly efficient in this activity. By testing various fragments of the MK-rich domain, we identified a 49 amino-acid-long sequence, designated Fr1, that could largely recapitulate the Plg-binding ability of whole PbsP. Fr1 constitutes the major portion of the MK-rich domain and spans a region in which methionine and lysine residues make up 21 and 17%, respectively, of the entire amino acid sequence. The presence of positively charged amino acids, such as lysine, in the context of hydrophobic residues, such as methionine, is a common feature of Plg binding motifs (Peetermans et al., 2016). Moreover, lysine residues in these motifs are often crucially required for the ability of either host- or pathogen- derived proteins to bind Plg (Miles et al., 1991; Ponting et al., 1992; Bergmann et al., 2001; Derbise et al., 2004; Wiles et al., 2010; Bhattacharya et al., 2012; Sanderson-Smith et al., 2012; Moreau et al., 2017; Nagarajan et al., 2019). For these reasons we hypothesized that one or more of the lysine residues of this domain mediated such interactions. Surprisingly, however, this was not the case, since mutant forms of the MK-rich domain lacking one or all lysine residues fully retained the ability to bind Plg, as shown here by direct binding or inhibition studies. These data are reminiscent of recent studies in which mutagenesis of lysine residues to alanine resulted in minimal or no inhibition in Plg binding activity. For example, loss of lysine residues in the internal nonapeptide of a1 and a2 repeats of the M-like proteins of *S. pyogenes* did not decrease its Plg binding affinity. However, Plg binding was abolished by mutagenesis of arginine and histidine residues, despite the presence of lysine residues (Sanderson-Smith et al., 2006; Sanderson-Smith et al., 2007; Sanderson-Smith et al., 2012). Therefore it appears that the absence of lysine residues can be compensated for by the presence of other positively charged amino acids such as arginine or histidine within Plg binding motifs in these bacterial Plg receptors. It should be noted, however, that the MK-rich region of PbsP does not contain positively charged amino acids other than lysine. Therefore our data showing robust, lysine-inhibitable interactions between Plg and a mutated MK-rich fragment lacking all lysine residues provide an unusual example of an LBS ligand devoid of positively charged amino acid residues. We speculate that methionine residues in the MK-rich region of PbsP engage in hydrophobic interactions with aromatic residues in the in the LBS pocket of the K4 domain. Our data showing partial inhibition of these interactions in the presence of free L-methionine are compatible with this possibility.

In conclusion, we identified here a novel bacterial sequence that can interact with the LBS of Plg even in the absence of lysine or other positively charged amino acids. These data may be useful to devise alternative therapeutic strategies to prevent Plg-mediated invasion of host tissues by GBS and other bacterial pathogens.

## Data Availability Statement

The original contributions presented in the study are included in the article/**Supplementary Material**. Further inquiries can be directed to the corresponding author.

## Author Contributions 

FC, LR and GP conducted most of the experiments and analyzed data. Other experiments were conducted by GL, GG and RG. CB and GT contributed to the study design, wrote the paper and revised the final version of the manuscript. All authors contributed to the article and approved the submitted version.

## Funding

The work was funded in part by the PRIN (Programma di Ricerca Scientifica di Rilevante Interesse Nazionale) grant 2017M8R7N9_002 from the Ministero dell’Università and Ricerca Scientifica (MIUR) of Italy and by FFABR (Fund For Basic Research Activities) 2017 and 2019 from the University of Messina. Additional funding was provided by a grant for PhD student (PON n° DR_36_TRAN_PON_IND_1) at Doctoral School in Translational Molecular Medicine and Surgery, Department of Biomedical, Dental and Imaging Sciences, University of Messina, Messina, Italy.

## Acknowledgments

We thank Dr. Patrick Trieu-Cuot from Institute Pasteur, Paris, France, for helpful discussion and suggestions.

## Conflict of Interest

CB and GT act as scientific advisors for, respectively, Scylla Biotech Srl. and Charybdis Vaccines Srl. without receiving any compensation for these activities. Charybdis Vaccines S.r.l. and Scylla Biotech S.r.l. did not provide funding for this study and had no role in its conduction.

The remaining authors declare that the research was conducted in the absence of any commercial or financial relationships that could be construed as a potential conflict of interest.

## Publisher’s Note

All claims expressed in this article are solely those of the authors and do not necessarily represent those of their affiliated organizations, or those of the publisher, the editors and the reviewers. Any product that may be evaluated in this article, or claim that may be made by its manufacturer, is not guaranteed or endorsed by the publisher.
