# Supplementary material for: Lysine Residues in the MK-Rich Region Are Not Required for Binding of the PbsP Protein From Group B Streptococci to Plasminogen
Source: Front Cell Infect Microbiol. 2021 Sep 8;11:679792. doi: 10.3389/fcimb.2021.679792 (PMC8455988; doi:10.3389/fcimb.2021.679792)
Supplement: Supplementary file 1 [file DataSheet_1.pdf]

Table S1. Amino acid sequence of MK-rich domain

M12K (N_{423} – N_{484}):

NSTSMMM**KK**D**K**MTMPM**KK**EMTSS**K**INTGMMMSNN**K**MSANMQMSSQA**K**SND**K**AG**KK**MSMMS**K**N

Mut1 (**Lys430 Lys 431 Lys 433 Lys 439** and **Lys 440** replaced with **alanine**):

NSTSMMM**AA**D**A**MTMPM**AA**EMTSSKINTGMMMSNNKMSANMQMSSQAKSNDKAGKKMSMMSKN

Mut2 (**Lys446** and **Lys457** replaced with **alanine**):

NSTSMMMKKDKMTMPMKKEMTSS**A**INTGMMMSNN**A**MSANMQMSSQAKSNDKAGKKMSMMSKN

Mut3 (**Lys469 Lys 473 Lys 476 Lys 477** and **Lys 483** replaced with **alanine**):

NSTSMMMKKDKMTMPMKKEMTSSKINTGMMMSNNKMSANMQMSSQA**A**SND**A**AG**AA**MSMMS**A**N

Mut4 (**Lys430 Lys 431 Lys 433 Lys 439 Lys 440 Lys446 Lys457 Lys469 Lys 473 Lys 476 Lys 477** and **Lys483** replaced with **alanine**):

NSTSMMM**AA**D**A**MTMPM**AA**EMTSS**A**INTGMMMSNN**A**MSANMQMSSQA**A**SND**A**AG**AA**MSMMS**A**N

***** Lysine residues in the M12K fragment and mutated Lysine residues in the Mut 1-4 fragments are indicated in underlined bold.
