# Supplementary material for: Lysine Residues in the MK-Rich Region Are Not Required for Binding of the PbsP Protein From Group B Streptococci to Plasminogen
Source: Front Cell Infect Microbiol. 2021 Sep 8;11:679792. doi: 10.3389/fcimb.2021.679792 (PMC8455988; doi:10.3389/fcimb.2021.679792)
Supplement: Supplementary file 2 [file DataSheet_2.pdf]

Table S2. Plasmids and primers used in the study

I.D.	Description [a-b]
Plasmids	
pDONR221	*E. coli* cloning vector, Km^R , with attR sites for the Gateway cloning system
pDEST15	*E. coli* expression vector, Amp^R , with attR sites for the Gateway cloning system
pDEST15_M12K	pDEST15 vector carrying a sequence encoding M12K (residues 423 - 484)
pDEST15_Mut1	pDEST15_M12K vector encoding for Mut1
pDEST15_Mut2	pDEST15_M12K vector encoding for Mut2
pDEST15_Mut3	pDEST15_M12K vector encoding for Mut3
pDEST15_Mut4	pDEST15_M12K vector encoding for Mut4
Primers	
	To amplify oligonucleotides encoding for M12K and Mut1
$M12K_{423}$_up	GGGG**ACAAGTTTGTACAAAAAAGCAGGCTTT**AATAGCACATCAATGATGATG
$M12K_{484}$_low	GGGG**ACCACTTTGTACAAGAAAGCTGGG**TTTTAATTTTTAGACATCATAGACAT
	To amplify oligonucleotides encoding for Mut2, Mut3, Mut4
$M12K_{423}$_up	GGGG**ACAAGTTTGTACAAAAAAGCAGGCTTT**AATAGCACATCAATGATGATG
$Mut4_{483}$_low	GGGG**ACCACTTTGTACAAGAAAGCTGGG**TTTTAATTTGCAGACATCATAGACAT

a. Km^R , kanamycin resistance; Amp^R, ampicillin resistance.
b. Recombination sites are indicated by nucleotides in bold.
